# Convergence of Password Guessing to Optimal Success Rates †

**DOI:** 10.3390/e22040378

**Published:** 2020-03-26

**Authors:** Hazel Murray, David Malone

**Affiliations:** Department of Mathematics and Statistics and the Hamilton Institute, Maynooth University, R51 A021 Co. Kildare, Ireland; david.malone@mu.ie

**Keywords:** passwords, guessing, dataset, distribution

## Abstract

Password guessing is one of the most common methods an attacker will use for compromising end users. We often hear that passwords belonging to website users have been leaked and revealed to the public. These leaks compromise the users involved but also feed the wealth of knowledge attackers have about users’ passwords. The more informed attackers are about password creation, the better their password guessing becomes. In this paper, we demonstrate using proofs of convergence and real-world password data that the vulnerability of users increases as a result of password leaks. We show that a leak that reveals the passwords of just 1% of the users provides an attacker with enough information to potentially have a success rate of over 84% when trying to compromise other users of the same website. For researchers, it is often difficult to quantify the effectiveness of guessing strategies, particularly when guessing different datasets. We construct a model of password guessing that can be used to offer visual comparisons and formulate theorems corresponding to guessing success.

## 1. Introduction

The resistance of passwords to guessing is essential to our online security. Passwords are used to protect our online banking, shopping, insurance accounts, social media, and email, among many other things. An attacker’s goal is to gain access to these accounts, often by directly guessing passwords. Once an attacker has access, they can make a financial gain from that user’s account directly or use the user as a base for spam, botnet, ransomware, and phishing attacks. Our work in this paper is important to gain a better understanding of our vulnerabilities to password attackers and to raise the awareness of organizations to the damage leaked password datasets can cause. This paper is an extension of work presented in Reference [1].

Guessing passwords in the right order is important for an attacker as they wish to compromise as many users as possible with a small number of guesses. Password guessing can be divided into two types: online guessing and offline guessing. In an online guessing attack, the attacker attempts to guess combinations of the username and password directly on the live system, for example, on a website login page. The number of guesses is often limited by throttling techniques, such as locking a user out when a certain number of wrong guesses has been reached. In this case, an attacker will often need to make a correct guess in less than, say, 100 attempts [2].

An offline guessing attack can only occur after a dataset of passwords has been leaked. These leaks occur relatively frequently and can result in reputational damage to multinational organizations and governments [3]. Once a password dataset has been leaked, then the method that was used to store the passwords is important. If passwords are stored in plaintext or they are encrypted and the key is leaked, then they are already available to attackers. If they are hashed and unsalted, then most passwords can be found using a hash lookup table (rainbow tables) [4]. Finally, if they are hashed and salted, then offline guessing is necessary [5]. In an offline guessing attack, the attacker guesses a password, combines it with the random salt associated with that user, and then hashes the combined value. The attacker compares this hash to all the hashes in the leaked dataset to see if any users chose this guessed value as their password. The goal of the attacker is to compromise as many users as possible with as few guesses as possible and, therefore, get the best returns for their time and resources used.

Attackers will often use large dictionaries of words, as well as lists of most popular password choices to inform their password guesses. It is important for researchers to understand the methods used by attackers in order to know how to best protect online accounts, and there has been considerable research in this area [6,7,8,9,10,11]. Melicher et al. modeled password guessability using neural networks [6], Durmuth et al. used an ordered Markov enumerator to simulate attackers’ password guessing [7], Hitaj et al. used deep learning [8], and Weir et al. used probabilistic context-free grammars [9]. Ur et al. investigated the effectiveness of automated cracking programs, such as John the Ripper and Hashcat, and found that automated guessing is less effective than real-world cracking by professionals [12]. Li et al. studied the differences between passwords from Chinese and English users [13]. Wei et al. [14] and Malone and Maher [15] found that the choice of passwords often reflects the service the password is chosen for. Castelluccia et al. discovered that including the users’ personal details in password guessing improves success rates [16]. Work on the distribution of passwords has also been investigated. Researchers have compared password choices to a Zipf distribution [15,17], and Bonneau [18] looked at a number of different options for password guessing metrics.

In this paper, we formalize an understanding of password guessing success. This allows a comparison of guessing success and is particularly useful for comparing guessing when a variety of password datasets are used. This formulation of guessing success also allows for the development of a model of guessing that can be analytically studied. We use it to prove that convergence to optimum guessing success rates occurs when a sample of passwords is used to guess a whole dataset. We continue by showing empirically that this convergence occurs when real leaked password datasets are used. In fact, we can show that, when a sample of users are compromised, information is also revealed of the characteristics of the remaining users’ passwords, thus allowing us to see an effect on the guessing success when informing our guessing using the leaked sample.

In Section 2, we introduce a model for measuring the effectiveness of password guessing. In Section 3, we prove that, using a sample of passwords, we can effectively guess the password in a dataset with a loss that converges to zero as the sample becomes large. Section 4 provides a graphical example of the guessing function and introduces real-world leaked datasets. In Section 5, we use our guessing function to demonstrate the effectiveness of samples at compromising the remaining users in the dataset from which they were drawn. Section 6 introduces potential variants of our convergence theorems that allow for a small amount of guessing loss and, therefore, better describe the empirical data and the motivations of an attacker. Finally, in Section 7, we demonstrate the threat to an organization of a subset of their users being compromised. We show that organizations are vulnerable if a subset of their passwords is leaked.

## 2. Model

When we report the number of users that a guessing technique compromises, it is important to consider this in light of the maximum number of users it is possible to compromise for that number of guesses on that dataset. This can depend on the total number of users in the dataset and also on the distribution of password choices by those users. Therefore, reporting the number of guesses that a technique makes does not give the full picture unless context is provided. We, therefore, begin by introducing a model that can report effective guessing measured with respect to the guessing potential.

Suppose we have a set of passwords *X* chosen by *N* users. We rank and order these passwords, so the most popular password is rank 1, the second most popular is rank 2, and so on, until rank |X|, where |X| is the number of different password choices; we can break ties between equally popular passwords arbitrarily. Let p(x) be the probability that the password *x* is used by a randomly selected user from the group *N*; note here that *p* will be a probability distribution, and we can extend it to a probability measure on *X*. Let σ(k) be the password of rank *k* in distribution *p*.

*Question: If we take n samples from a dataset X of users’ passwords, how effectively do these n samples guess the passwords of the other N users from the same dataset?*

First, we define a method for measuring the effectiveness of guessing.

### 2.1. Optimal Guessing

The optimal guessing strategy [19] involves using the rank and order of *p*. If we guess the first *g* passwords, then the fraction of passwords guessed is

(1) $$F\left(g\right)=\sum_{k=1}^{g}p\left(\sigma \left(k\right)\right).$$

The function is cumulative because we want to know after *g* guesses how many users we have compromised, rather than caring about how many users were compromised on the *g*-th guess.

### 2.2. Guessing with a Sample

Suppose we take a sample of *n* passwords and rank and order them to form a second distribution $q^{n}$. Let $\sigma_{q^{n}}\left(k\right)$ be the rank of password *k* in the distribution $q^{n}$. Now, we use the distribution $q^{n}$ to guess passwords that are actually distributed with frequency *p*. We can define a function similar to the one above that will tell us how many users in *p* will be compromised when using the rank and order of the passwords in $q^{n}$.

(2) $$G_{q^{n}}\left(g\right)=\sum_{k=1}^{g}p\left(\sigma_{q^{n}}\left(k\right)\right).$$

### 2.3. Guessing Loss

Combining the optimal guessing strategy with guessing using a sample, we define a method for describing how well a set of ordered password guesses can guess a dataset of passwords as:

(3) $$H_{q^{n}}\left(g\right)=F\left(g\right)-G_{q^{n}}\left(g\right)=\sum_{k=1}^{g}p\left(\sigma \left(k\right)\right)-p\left(\sigma_{q^{n}}\left(k\right)\right).$$

This function measures the gap between the optimal strategy and the ability of a probability distribution $q^{n}$ to guess a second probability distribution *p*.

## 3. Proof of Convergence of Password Guessing

Using this model, we might hope to use a sample to drive password guessing with zero loss. When $H_{q^{n}}\left(g\right)\equiv 0$, we have guessed every user’s password as fast as possible. We will now prove that, as the number of samples increases, we converge towards a zero-loss system.

**Definition** **1** (δ). *Consider the minimum size of the gap between two distinct adjacent probabilities of p:*

$$\delta =\min_{g}\left\{p(\sigma (g))-p(\sigma (g+1))\;|\;p(\sigma (g))\neq p(\sigma (g+1))\right\},$$

*where we take p(σ(g))=0 if g>|X|.*

**Lemma** **1.** *Suppose $q^{n}$ is a sample of size n drawn from the distribution p. A lower bound on the probability of zero loss can be given by*

$$P\left[H_{q^{n}}=0\right]\geq P[∥p-q^{n}{∥}_{\infty }<\delta /2].$$

**Proof.**  Consider probability distributions *q*, where ${∥p-q∥}_{\infty }<\delta /2$. Note that, for the two passwords *w* and $w^{\prime }$, if we had $p\left(w\right)>p\left(w^{\prime }\right)$, then $q\left(w\right)>q\left(w^{\prime }\right)$, as the difference between p(w) and $p(w^{\prime })$ must have been at least δ, but |p(w)−q(w)|<δ/2, and likewise for $w^{\prime }$. Thus, $\sigma_{q}$ orders passwords of differing *p* probability in the same order as *p*.

On the other hand, if $p\left(w\right)=p\left(w^{\prime }\right)$, and *w* is replaced by $w^{\prime }$ in the ordering $\sigma_{q}$, then the contribution to $H_{q^{n}}$ is $p\left(w\right)-p\left(w^{\prime }\right)=0$. We conclude that if ${∥p-q∥}_{\infty }<\delta /2$, then $H_{q^{n}}=0$. ☐

We will now show that the second probability in Lemma 1 goes to 1 as n→∞ using Sanov’s Theorem.

**Theorem** **1** (Convergence to optimal guessing using Sanov’s theorem)**.** *Suppose $q^{n}$ is a sample of size n drawn from the distribution p.*

$$P\left[H_{q^{n}}=0\right]\geq P[∥p-q^{n}{∥}_{\infty }<\delta /2]\to 1\;as\;n\to \infty$$

*and*

$$P[∥p-q^{n}{∥}_{\infty }<\delta /2]\geq 1-{\left(n+1\right)}^{\left|X\right|}2^{-n\frac{\delta^{2}}{2}}.$$

**Proof.**  Let *A* be the set of *q* with ${∥p-q∥}_{\infty }\geq \delta /2$. Pinsker’s inequality tells us that, for any *q*,

$$\max_{E\subset X}\{|p\left(E\right)-q\left(E\right)|\}\leq \sqrt{\frac{D_{KL}\left(q||p\right)}{2}},$$

where *E* is any event. In particular, if q∈A, then the gap between the probabilities is always at least δ/2, and the max on the left must be at least δ/2, so

$$\frac{\delta }{2}\leq \sqrt{\frac{D_{KL}\left(q||p\right)}{2}},$$

and so $\delta^{2}/2\leq D_{KL}\left(q||p\right)$. Sanov’s Theorem says

$$P\left[q^{n}\in A\right]\leq {\left(n+1\right)}^{\left|X\right|}2^{-nd^{*}},$$

where

$$d^{*}=\min_{q\in A}D_{KL}\left(q||p\right).$$

$$\Rightarrow P\left[q^{n}\in A\right]\leq {\left(n+1\right)}^{\left|X\right|}2^{-n\frac{\delta^{2}}{2}}.$$

We conclude that $P[q^{n}\in A]\to 0$, so $P[∥p-q^{n}{∥}_{\infty }<\delta /2]\to 1$ as n→∞. ☐

Theorem 1 shows that a sample of size *n* will converge to the distribution of the whole dataset *p* with a probability approaching 1 as n→∞.

However, this convergence is slow. The turning point of the probability bound is

$$\frac{\delta^{2}\ln2}{2}-\frac{\left|X\right|}{n+1}=0.$$

This tells us the point at which the function must start increasing towards 1. It is determined by the ratio of the sample size *n* to the total size of the dataset |X|. Rearranging, we can find the value of *n* that the bound must have turned by is

$$n=\frac{2|X|}{\delta^{2}\ln2}+1.$$

Since δ is a probability value between 0 and 1, we can see that the number of samples *n* in *q* will need to be at least two times greater than the number of different passwords in the dataset.

In fact, typical values of δ will result in considerably larger *n*. δ relates to the difference in frequency between two adjacently ranked passwords. This difference in frequency is often 1. Given a dataset of *N* users, and a probability difference between a password chosen by two users and a password chosen by one user of δ=1/N, this will give

$$n=\frac{2|X|N^{2}}{\ln2}+1.$$

While Theorem 1 does demonstrate that our guesses success will converge to zero, the rate of convergence is too slow to be useful. We, therefore, present a second method for identifying an alternative lower bound for the convergence.

**Theorem** **2** (Convergence to optimal guessing using the central limit theorem)**.** *Suppose $q^{n}$ is a sample of size n drawn from the distribution p. Then, as n→∞*

$$P\left[H_{q^{n}}=0\right]\geq P[∥p-q^{n}{∥}_{\infty }<\delta /2]\geq {\left(1-Q\left(\frac{\delta \sqrt{n}}{{2∥A∥}_{\infty }}\right)\right)}^{\left|X\right|}\to 1,$$

*where Q(x) is the probability that a standard normal random variable takes a value larger than x.*

**Proof.**  Given that $q^{n}$ is sampled from distribution *p* in an essentially multinomial way, we know that $E[q^{n}]=p$ and that the covariance for the entries of $q^{n}$ will be Σ/n. Where

$$\Sigma =\left\{\begin{array}{cc} p_{i}\left(1-p_{i}\right) & \mathrm{if}\;i=j\\ -p_{i}p_{j} & \mathrm{otherwise}. \end{array}\right.$$

The multivariate central limit theorem tells us that $q^{n}$ is approximately normal N(p,Σ/n).

Note that Σ is real valued, symmetric, and diagonally dominant, and so positive semidefinite, thus having non-negative eigenvalues. Consequently, by spectral decomposition, we can write $\Sigma =U\Lambda U^{T}$, where $UU^{T}=I$ and Λ is the matrix with Σ’s eigenvalues on the diagonal. Let $A=U\Lambda^{1/2}$, and then $AA^{T}=\Sigma$ and samples from N(p,Σ) can be generated by forming a vector *z* with coordinates that are N(0,1) and then taking $p+Az/\sqrt{n}$.

Now,

$$P\left[∥p-q^{n}{∥}_{\infty }<\frac{\delta }{2}\right]=P\left[\frac{{∥Az∥}_{\infty }}{\sqrt{n}}<\frac{\delta }{2}\right]\geq P\left[\frac{{∥A∥}_{\infty }{∥z∥}_{\infty }}{\sqrt{n}}<\frac{\delta }{2}\right]=P\left[{∥z∥}_{\infty }<\frac{\delta \sqrt{n}}{{2∥A∥}_{\infty }}\right].$$

Or, in terms of the *Q* function, noting that *z* has |X| independent components,

$$P\left[{∥z∥}_{\infty }<\frac{\delta \sqrt{n}}{{2∥A∥}_{\infty }}\right]={\left(1-Q\left(\frac{\delta \sqrt{n}}{{2∥A∥}_{\infty }}\right)\right)}^{\left|X\right|}.$$

So, as n→∞, Q(.)→0, so P[.]→1. ☐

In fact, to simplify this expression, we can give an estimate for ${∥A∥}_{\infty }$. The entries of *A*, $a_{ij}=u_{ij}\lambda_{j}^{1/2}$, and so by the Cauchy–Schwartz inequality,

$${∥A∥}_{\infty }\leq ∥u_{i}{∥}_{2}\;{∥\lambda^{1/2}∥}_{2}.$$

However, $∥u_{i}{∥}_{2}=1$ because the rows of *U* are orthogonal vectors and

$$∥\lambda^{1/2}{∥}_{2}=\sqrt{\sum_{j}{\left(\lambda_{j}^{1/2}\right)}^{2}}=\sqrt{\mathrm{trace}(\Sigma )}.$$

So,

$${∥A∥}_{\infty }\leq \sqrt{\mathrm{trace}(\Sigma )}=\sqrt{p_{1}\left(1-p_{1}\right)+p_{2}\left(1-p_{2}\right)+\ldots }.$$

Note that, as $\sum p_{i}=1$, we can find the largest possible value of $p_{1}\left(1-p_{1}\right)+p_{2}\left(1-p_{2}\right)+\ldots$ to be 1−1/|X|≤1.

We conclude that

$$P\left[∥p-q^{n}{∥}_{\infty }<\frac{\delta }{2}\right]\geq {\left(1-Q\left(\frac{\delta \sqrt{n}}{{2∥A∥}_{\infty }}\right)\right)}^{\left|X\right|}\geq {\left(1-Q\left(\frac{\delta \sqrt{n}}{2\sqrt{\mathrm{trace}\Sigma }}\right)\right)}^{\left|X\right|}\geq {\left(1-Q\left(\frac{\delta \sqrt{n}}{2}\right)\right)}^{\left|X\right|}.$$

Unlike our previous bound, this bound derived by the central limit theorem is monotonic in *n*. The convergence function is monotonic and between 0 and 1. The parameters |X| and *n* determine the rate of convergence but there is no turning point of the function. This function offers a better bound on the convergence.

**Figure 1:**
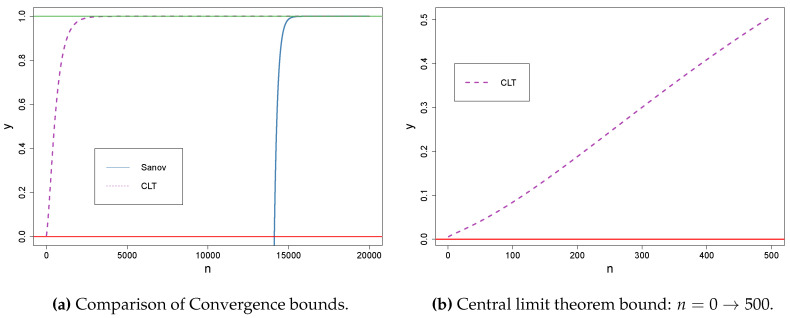
(**a**) Convergence bound with Sanov’s theorem versus with (**b**) the central limit theorem.

For example, take a toy dataset with just 8 users and a minimum distance between the probability of password choices as δ=1/N=0.125. Figure 1a shows the convergence to 1 for both bounds. Even for this small dataset size, the number of samples needed for a probability of close to 1 is large. We can see the bound provided by the central limit theorem is much better than the Sanov bound. This effect is further emphasized the larger the size of the dataset *X*.

## 4. Test on Real-World Leaked Password Datasets

Theorems 1 and 2 tell us that, if we continue to choose samples from a dataset, then these will have zero guessing loss with probability approaching 1 as n→∞. We are interested in the application of this convergence to a real-world password leak. Our question is whether a leak of a subset of a dataset can, in practice, give away valuable information about the distribution of the remaining passwords in that same dataset.

In this section, we introduce five datasets of passwords that have been leaked to the public from real organizations. We comment on the distribution of these passwords. Then, sampling passwords from these datasets, we see whether the samples provide an effective guessing strategy with respect to our guessing loss function.

### 4.1. Datasets

#### 4.1.1. Computerbits.ie Dataset: N=1795

**Figure 2:**
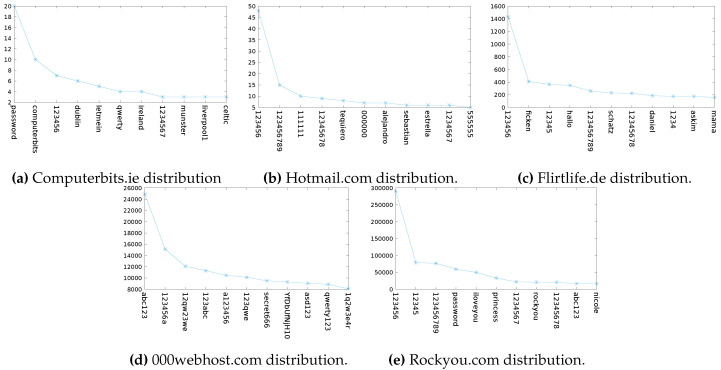
Distribution of password choices: frequency of passwords in ranks 1 to 11.

In 2009, 1795 users’ passwords were leaked from the Irish website Computerbits.ie. Figure 2a shows the top 11 most popular passwords in this dataset. We can see many Irish-orientated words: dublin, ireland, munster, celtic. The second most popular password for the website Computerbits.ie was “computerbits”, reinforcing the idea that the service provider has an impact on the user’s choice of password [14].

#### 4.1.2. Hotmail.com Dataset: N=7300

Ten thousand users’ passwords from the website Hotmail.com were made public in 2009 when they were uploaded to pastebin.com [20] by an anonymous user. Though it is still unknown, it is suspected that the users had been compromised by means of phishing scams [21]. Figure 2b shows the frequency of the first 11 most popular passwords chosen by users in the Hotmail dataset. The most popular password is “123456” which occurs with frequency 48. The password of rank 11 occurs with frequency 5. So, all passwords of rank greater than ten have a frequency less than or equal to 5. In total, there are 6670 ranks in the Hotmail distribution, i.e., there are 6670 distinct passwords in the dataset. The top password represents 0.66% of the Hotmail users’ passwords, and the rank 11 password represents 0.068% of the Hotmail users’ passwords.

#### 4.1.3. Flirtlife.de Dataset: N= 98,912

In 2006, over 100,000 passwords were leaked from a German dating site Flirtlife.de. A write-up by Heise online [22], a German security information website, states that the leaked file contained many usernames and passwords with typographic errors. It seems that attackers were harvesting the data during log-in attempts.

This means that, in the dataset, for a small number of users, we had multiple passwords. We followed the clean up method specified in Reference [15]: we took the user’s password as the last entry seen for that user. In this way, we ignore passwords where the users first attempts were the wrong password or previous passwords when a password change has occurred. There were also 18 blank password fields, and we removed these from the dataset, too. After this cleaning process, we were left with 98,912 users and 43,838 different passwords.

The distribution of the first 11 passwords from the Flirtlife dataset are shown in Figure 2c. As noticed by Reference [15], we see that passwords in these top ranks have meanings in German or Turkish. For example, ‘schatz’ and ‘askin’ are German and Turkish terms of endearment, respectively. The rank 1 password represents 1.45% of the Flirtlife users’ passwords, and the rank 11 password represents 0.16% of the Flirtlife users’ passwords.

#### 4.1.4. 000webhost.com Dataset: N= 15,252,206

In 2015, 15 million users’ passwords were leaked from 000webhost.com [23]. The attacker exploited a bug in an outdated version of PHP. The passwords were plaintext and created with a composition policy that forced them to include at least 6 characters which must include both letters and numbers. Today, a lot of websites will enforce a composition policy on passwords. It will be interesting to see the impact this has on the effectiveness of guessing the password dataset distribution. The leaked dataset was cleaned in the same way as in Reference [23]. All passwords longer than 256 characters and passwords that were not ASCII were removed.

The distribution of the top 11 passwords in the 000webhost.com dataset is shown in Figure 2d. All passwords are a mixture of letters and numbers. There is a clear pattern to each password, though less obviously for the password ‘YfDbUfNjH10305070’. The letters, YfDbUfNjH, can be mapped to a Russian word which means “navigator” [11]. It is unclear why this password is so popular. It could be a Russian botnet that is using the same password for each of its bot accounts [24]. There are 10 million distinct passwords in the dataset. The rank 1 password represents a surprisingly low 0.16% of the users’ passwords, and the rank 11 password represents 0.05%.

#### 4.1.5. Rockyou.com Dataset: N= 32,602,877

In December 2009, 32 million user credentials were leaked by the company Rockyou. The passwords were stored in plaintext and the hackers used a 10-year-old SQL vulnerability to gain access to the database. The platform did not allow the inclusion of special characters in passwords. Figure 2e shows the distribution of the first 11 Rockyou passwords. The rank 1 password in the Rockyou dataset represents 0.89% of the total dataset, and the rank 11 password represents 0.05% of the total users’ passwords in the dataset.

### 4.2. Demonstration of Guessing Function

In Section 2.3, we defined a method for measuring the effectiveness of password guessing. We will now graph the functions $F_{p}\left(g\right)$, $G_{q^{n}}\left(g\right)$ and $H_{q^{n}}\left(g\right)$. Note that the *y*-axis for functions $F_{p}\left(g\right)$, $G_{q^{n}}\left(g\right)$ is a measurement of number of successes and therefore we want large values. But, $H_{q^{n}}\left(g\right)$ is a measurement of cumulative loss, so we want $H_{q^{n}}\left(g\right)$ to be small. The goal is to have the sample guessing, $G_{q^{n}}\left(g\right)$, as close as possible to the optimal guessing, $F_{p}\left(g\right)$, thus keeping the loss, $H_{q^{n}}\left(g\right)$, as low as possible.

**Figure 3:**
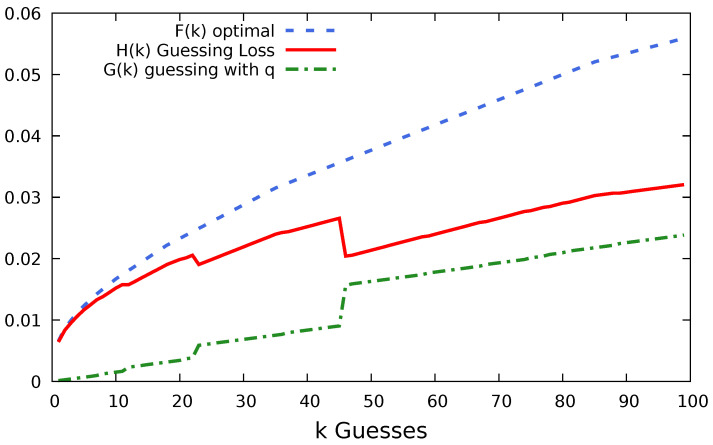
Hotmail dataset guessed using a sample of size n=100 users.

In Figure 3, we chose n= 100 users’ passwords, with replacement, from the Hotmail dataset. The 100 users’ passwords were ranked and ordered and used to guess the passwords of all the 7300 Hotmail users whose passwords are in our dataset. We were able to compromise a total of 234 users, 134 more than those in the original sample.

The sample of 100 users was chosen randomly from the 7300 users in the Hotmail dataset. But, because we sample with replacement, we managed to choose the same user twice. We discover this because there were 99 guesses made instead of 100, but the password of frequency 1 in *q* resulted in only 1 success in *p*. Note that, because all passwords after rank 1 in *q* occur with frequency 1, the ordering is arbitrary. Therefore, in this example, the jumps in loss could occur at any point after guess 1.

We can see a small reduction in loss at guess number 23, where 15 users were compromised. This was when the second most popular password “123456789” was guessed. On guess 46, the most popular password in the Hotmail dataset was guessed. This guess of password “123456” compromised 48 users.

Even for this small sample size (only 1% of the total dataset), we have a low loss. We only failed to compromise 0.03 of the users we could have potentially compromised with the optimum first 100 guesses. $G_{q^{n}}\left(g\right)$ achieved almost half the efficiency of the optimal strategy with 100 guesses.

## 5. Empirical Evidence of Convergence

Our convergence proof, Theorem 1, leveraging Sanov’s Theorem tells us that the convergence of the loss function goes towards zero with respect to $\frac{\left|X\right|}{n+1}$. That is, an important value for convergence is the ratio of the number of different passwords in the full dataset over the number of users’ passwords in the sample.

**Figure 4:**
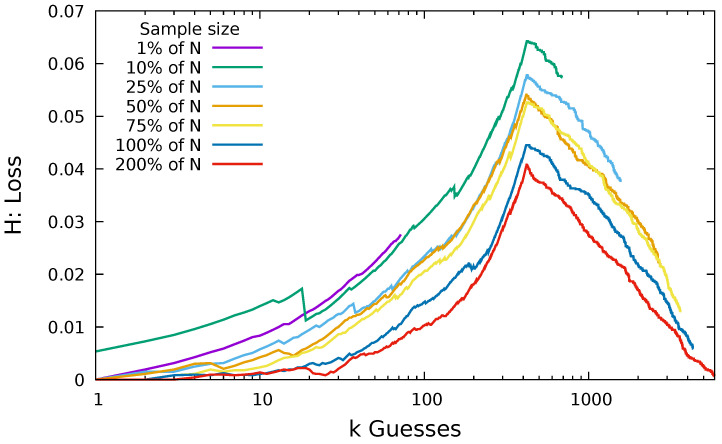
Probability loss: using samples of users from the Hotmail dataset to guess the passwords of all *N* users in the Hotmail dataset.

In this way, the larger the sample size, the smaller the loss we expect. In Figure 4, we take samples of size 1%, 10%, 25%, 50%, 75%, 100%, and 200% of the original dataset size. For example, a sample of 200% of the Hotmail dataset involves drawing from the 7300 users’ passwords 14,600 times with replacement.

As expected, we found that the increase in the number of samples does reduce loss. We initially see an increase in loss as the number of guesses increases. This increase occurs because we are dealing with a cumulative function, and the more guesses we make, the greater the potential we have to diverge from the optimal guessing. Notice that a peak occurs at approximately 420 guesses. In the Hotmail dataset, there are only 420 non-unique passwords (i.e., 420 password choices were chosen by more than one user). So, passwords in the Hotmail dataset after rank 420 have frequency 1; therefore each guess can only be equal to or more successful than the optimal at that rank.

**Figure 5:**
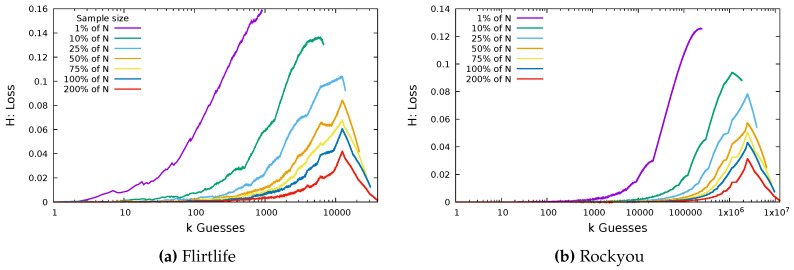
Probability loss for sample sizes n determined as a proportion of *N*.

The Hotmail dataset has a relatively large number of unique passwords. The ratio of number of users *N* to number of passwords |X| for the Hotmail dataset is 0.91, and 0.86 of the users chose a unique password. The ratio of users to passwords in the Flirtlife dataset is 0.44, and 0.32 of the users chose unique passwords. The ratio for the Rockyou dataset is also 0.44, and the fraction of users who chose unique passwords is 0.3645. In Figure 5, we plot the same loss function for the same proportions of the dataset, using the Flirtlife dataset and the Rockyou dataset.

Again, we see the larger sample sizes result in smaller loss. The largest value of loss for both was from the sample of size 1%. This resulted in a loss of 0.16 for Flirtlife and 0.125 for Rockyou, much higher than the largest Hotmail loss. Though it is not shown in the graphs, we note that the maximum loss for a sample from the Computerbits dataset was 0.055 and the maximum loss for a sample from the 000webhost dataset was 0.12. The turning point in the loss function occurs at approximately 12,442 guesses for Flirtlife. Twelve thousand, four hundred and forty-two is the number of non-unique passwords in the Flirtlife dataset. Similarly, we see the turning point for the Rockyou plots at ~2,450,000, matching the number of unique passwords in the Rockyou dataset.

In all three graphs, we can see that guessing using the sample is effective. When guessing using a sample, we lose, at most, 16% of the total users we could have compromised with the same number of optimal guesses. The loss of 16% for Flirtlife is the maximum over the *entire* range and with a sample of just 1% of the total number of users. If we look at the first 100 guesses when we have a sample of 10% of the users, we can see our loss is down at 0.007; that is, we are unable to compromise just 0.7% of the potentially compromisable users.

Figure 4 and Figure 5 show that we were effectively able to guess users’ passwords using a sample of passwords from other users of the same website. Even with a sample representing just 1% of the total number of users, we have a loss less than 0.16.

### 5.1. Spread of Results

In each of the previous graphs, we have only plotted results for one sample set of each size. But, results could vary depending on passwords chosen in the sample. Because we are anticipating convergence as n→∞, we expect the spread to decrease for increases in sample sizes.

**Figure 6:**
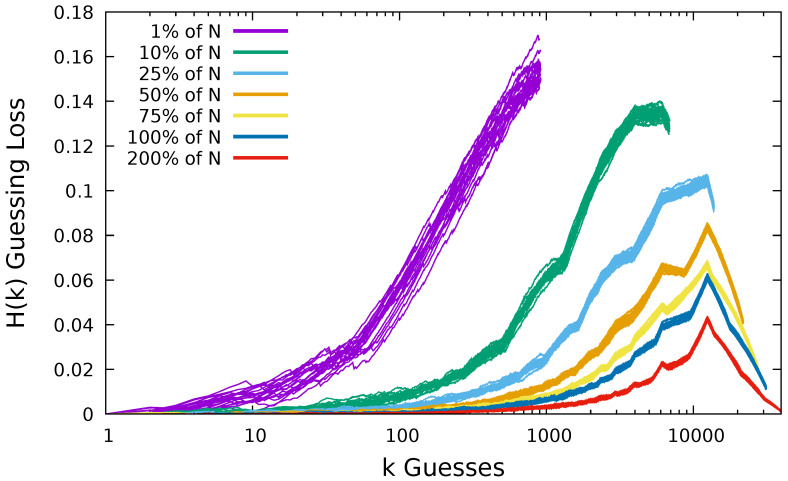
Guessing Computerbits.ie dataset: size N=1795.

In Figure 6, we plot the results of guessing the Flirtlife dataset using multiple samples of each size. We plot 20 different samples for each sample size. We can see that for a sample of size 1% of the dataset we have more spread in the data than for a sample 50% the size of the dataset and much more than for the sample of size 200%. At the end of the guessing, the difference between the max and min value for the samples of size 1% was just 0.021, representing a difference in users compromised of 2121. In Table 1, we summarize the spread of the results for guessing the Flirtlife dataset. We report these results as frequency loss, i.e., the number of users from N= 98,912 that we were unable to compromise.

### 5.2. When Does Loss Reach Zero?

Despite the fact that we have low loss, in none of these graphs do we reach a loss of zero. In Rockyou, for the sample of size 200%, we still had 26,795 users to compromise. Flirtlife we had 130 users, and Hotmail we had 5 users left to compromise.

Focusing on Hotmail, we investigated how many guesses it takes for our loss function to reach zero. Using a sample 300% the size of the number of users, we converge to zero with guess number 6172. In Figure 1b, we had a dataset with just 8 users, and for both Sanov and Central Limit Theorem (CLT), at 300% of 8, n=24, we can see we are still only just above 0 probability of convergence.

For the Flirtlife dataset, incrementing our sample size by 100% of N each time, we did not get a loss of zero until we had a sample 600% the size of *N*. In this case, we had a loss of zero repeatedly within the first 100 guesses, but as the number of guesses increased, we had approximately 120 users whose passwords we could not guess. We returned to a loss of zero at guess number 43,098. For a sample 600% of 8 in Figure 1a, n=48, we still have a probability near to 0. This implies that our construction of the bounds in the convergence proofs are more strict than desirable for representing real-world guessing effectiveness.

## 6. Improvements to Models

For the Flirtlife dataset, we need a sample 600% the size of the number of users in the set in order to get a loss of 0. But, with a sample size that is 50% the number of users in the dataset, we have a loss of just 4%. An attacker is unlikely to care about getting every single user and will likely be interested in compromising the most users with a reasonable number of guesses. In Theorems 1 and 2, we show that the loss will converge to zero as n→∞. But, instead, we could look at a situation where we just want our loss to be small. We now provide additional theorems that show that we can limit the amount of loss we are willing to accept.

Specifically, we want to show that, if we take *n* samples drawn from a distribution *p* of passwords and form the resulting distribution $q^{n}$, then as n→∞ we have $P[H_{q^{n}}<\epsilon ]\to 1$, i.e., in our guessing effectiveness we can choose to accept a small amount of loss, ϵ. We consider two methods for accepting an ϵ amount of loss. The first is a cut-off method, and the second is a blocking method.

### 6.1. Cut-Off Point to Allow ϵ Loss

**Figure 7:**
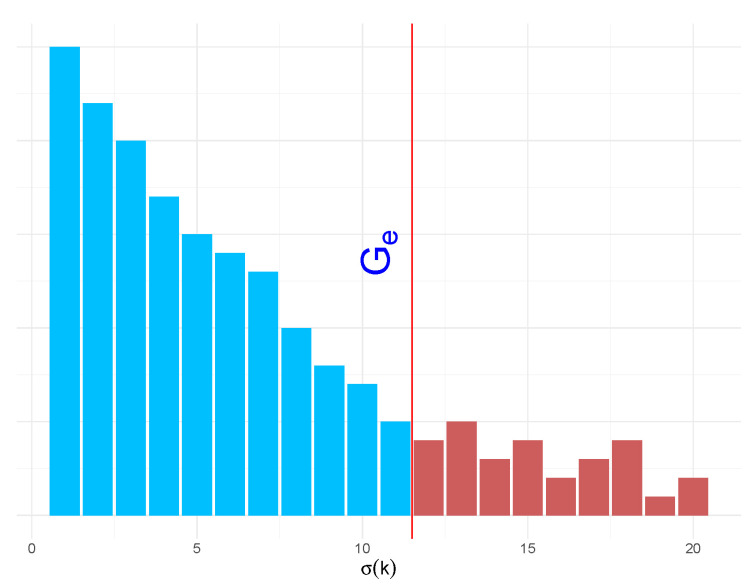
Demonstration of the cut-off point $G_{\epsilon }$.

The premise of this method is to divide the guessing into two parts, as shown in Figure 7. We consider the first part to be the high frequency passwords. For these, we want the guessing to be exact, i.e., the order of *q* matches the order of *p* for these ranks. After some cutoff point $G_{\epsilon }$, we are not particularly concerned with the order of the remaining passwords. We wish to define the cut-off point such that the loss does not exceed a value ϵ.

**Lemma** **2.** *The lower bound on the probability of ϵ loss is defined by*

$$P[||H_{q^{n}}{\left|\right|}_{\infty }<\epsilon ]\geq P[∥p-q^{n}{∥}_{\infty }<\delta_{\epsilon }/2],$$

*where $G_{\epsilon }$ is chosen such that*

$$\sum_{g=0}^{\lfloor n/2\rfloor -1}p\left(\sigma \left(G_{\epsilon }+g\right)\right)-p(\sigma (|X|-g))<\epsilon .$$

**Proof.**  When $g<G_{\epsilon }$, we proceed in the same way as in Lemma 1 to achieve zero loss, i.e., we choose

$$\delta_{\epsilon }=\min_{g<G_{\epsilon }}\left\{p(\sigma (g))-p(\sigma (g+1))\;|\;p(\sigma (g))\neq p(\sigma (g+1))\right\}.$$

Note that this $\delta_{\epsilon }$ will typically be larger than δ.

$$P\left[H_{q^{n}}{|}_{\left\{g<G_{\epsilon }\right\}}=0\right]\geq P[∥p-q^{n}{∥}_{\infty }<\delta_{\epsilon }/2].$$

When $g\geq G_{\epsilon }$, we know that the loss function H will be bounded by the case where *q* is ordered in the worst possible way. That is, the order of $q=p(\sigma (|X|)),\cdots ,p\left(\sigma \left(G_{\epsilon }\right)\right)$, where σ(|X|) is the last ranked password in *p*.

If there are *n* ranks between $\sigma (G_{\epsilon })$ and σ(|X|), then the loss will increase for at most the first ⌊n/2⌋ ranks; by the symmetry of $p(\sigma \left(G_{\epsilon }+g\right))$ and p(σ(|X|−g)). Therefore, the maximum value the loss can be is

$$\sum_{g=0}^{\lfloor n/2\rfloor -1}p\left(\sigma \left(G_{\epsilon }+g\right)\right)-p(\sigma (|X|-g))<\epsilon .\;☐$$

Using this method, we decide what value we are willing to accept as our ϵ loss and then work from there to compute the cut-off $G_{\epsilon }$. The assumption in this method is that an attacker is concerned with guessing the high frequency passwords in the exact order and cares less about the exact ordering of the low frequency passwords. This method can be employed to effectively model the actions of an attacker performing an online guessing attack.

### 6.2. Blocking Method to Allow ϵ Loss

In this method, we employ some leniency to allow small changes between the ranks of passwords. The assumption is that, if an attacker is making a block of 10 password guesses against all accounts, then it matters little to an attacker whether the rank 4 guess is more successful than the rank 2 guess because the loss will have balanced out after the 10 guesses.

Our motivation for this method is another attempt to increase the value δ. This should provide us with a stricter bound since an increase in δ will in turn increase $P[∥p-q^{n}{∥}_{\infty }<\delta /2]$, which bounds our convergence in Theorems 1 and 2.

**Figure 8:**
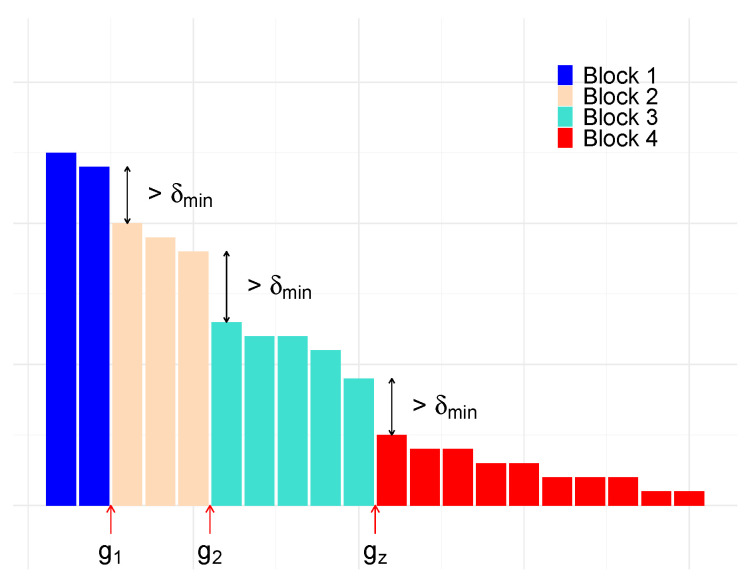
Demonstration of the δ block split.

The premise is that we can divide the passwords into blocks with the jump between each block of a defined size δ, as in Figure 8. We concede that all passwords can change order within the blocks, but no password can move outside the block, and the blocks cannot change order. The loss, ϵ, is a result of the passwords that we are unable to group into a block.

It is possible to maintain a model with a notional zero loss by mandating that passwords remaining outside the blocks are guessed with accuracy. However, by the logic that these will be the high ranks and, therefore, are likely to have low frequency, we decide not to assign any effort to correctly ordering these passwords and, instead, accept the loss. In the same way as for Lemma 2, we assume that such remaining passwords take the worst possible ordering; therefore, this provides us with a bound on our loss ϵ.

**Lemma** **3.** *Given a block of ranks separated by $\left\{g_{1},\cdots ,g_{z}\right\}$ and a $\delta_{min}>0$ so that*

$$p\left(\sigma \left(g_{i}\right)\right)-p\left(\sigma \left(g_{i}+1\right)\right)>\delta_{min},$$

*∀i={1,⋯,z}, we have*

$$P\left[H_{q^{n}}\left(g_{i}\right)=0\;\forall g_{i}\;and\;H_{q^{n}}\left(g\right)\leq \epsilon \;\forall g\;>\;g_{z}\right]\geq P[∥p-q^{n}{∥}_{\infty }<\delta /2].$$

**Proof.**  Take δ to be

$$\delta =\min_{1\leq i\leq z}\left\{p\left(\sigma \left(g_{i}\right)\right)-p\left(\sigma \left(g_{i}+1\right)\right)\right\}\geq \delta_{min}.$$

Then,

$$P\left[H_{q^{n}}\left(g_{i}\right)=0\right]\geq P[∥p-q^{n}{∥}_{\infty }<\delta /2].$$

The condition on the right ensures the blocks do not change order; so, at $g_{i}$, the end of a block, all passwords in previous blocks have been guessed, meaning $H_{q^{n}}\left(g_{i}\right)=0$.

If the last ranked password, σ(|X|), is contained inside the last block, then we have zero error. However, if we cannot create an end by using a jump of size $\delta_{min}$ for our last block, then those ranks outside of a block will denote our loss ϵ. This loss is bound by

$$\sum_{k=0}^{\lfloor n/2\rfloor -1}p\left(\sigma \left(g_{z}+k\right)\right)-p(\sigma (|X|-k))<\epsilon .\;☐$$

Note that this blocking method may have applications for certain types of password guessing. For most of our attacks, we assumed that passwords are guessed one at a time against all users, which is a reasonable match for some online guessing or offline guessing where an attacker has assigned a GPU core to each user/salt. Here, passwords could be guessed one at a time against each user by hashing them with respect to the user’s specific salt. However, another method of guessing could be to feed blocks of passwords to a GPU, with each core working on a different password. This might apply when password hashes were unsalted or all hashed with a common salt. In the latter case, a successful guess would happen when any password in a block matches; so, a model that disregards reordering within a block could be useful.

## 7. The Threat of Compromise From a Leaked Sample of Passwords

One of our initial claims was that revealing a sample of passwords from a dataset could help an attacker more than generic information about password choices. In this section, we provide evidence for this claim by using samples from one dataset to guess passwords in a different dataset. If there is a relationship between the passwords chosen by users at the same site, then we expect to get the lowest loss when guessing using passwords sampled from the same website.

### 7.1. Methodology

We took samples of size n=1000 users’ passwords from each of our four datasets and used these to guess the passwords of all the users in the full datasets. Below, we will discuss specific details regarding our methodology, and we then discuss results in Section 7.2.

#### 7.1.1. Sample Size

Our samples were size n=1000. It might have been desirable to use a larger sample size, for example, 10,000; however, we were limited by the number of passwords in the Computerbits password set and wanted the examples below to be comparable. In Section 5.1, we found that, for small sample sizes, there was a spread in the result of the guessing.

**Figure 9:**
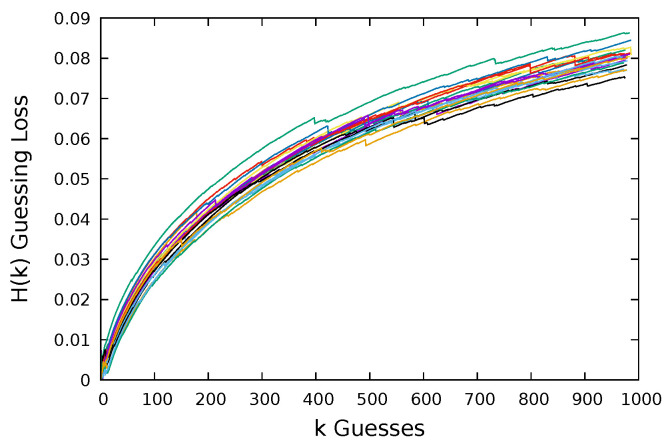
Rockyou 20 samples, size n=1000.

For example, in Figure 9, we plot the loss for 20 Rockyou samples of size n=1000. n=1000 represents just 0.003% of the Rockyou dataset; as a result, we see a diversity in the success of the sample at guessing. As a result of the potential for differing results, we will run multiple trials for each sample size.

#### 7.1.2. John the Ripper

We also included in the results the loss if the passwords are guessed using the top 1000 passwords in the John the Ripper (JtR) basic wordlist [25]. John the Ripper is one of many password cracking software tools. Its strength lies in its password mangling tools. It takes a base password and *mangles* it to create other related passwords. For example, it might start with the password ‘password’ and mangle it by exchanging vowels with symbols to create a password guess “p@ssw0rd”. For our comparison, we take the basic wordlist that JtR can work on, which has just 3546 words. In the classic configuration of JtR, these 3546 would be guessed and then word mangling rules would be applied to the words to produce subsequent guesses. By including the guessing success for JtR, we provide a reference point for the effectiveness of the guessing relative to this common tool. The first 10 passwords in the John the Ripper wordlist we used are listed in Table 2. These have similarities to the top 10 passwords in the leaked datasets, shown in Figure 2, but they cannot include website, topic, nor demographic-specific word choices.

#### 7.1.3. Sampling with and without Replacement

We present the results of guessing when we sample both with and without replacement. When we sample with replacement, we might consider that we have a false sense of success because we are counting successes when we compromise users whose passwords we already knew and were part of our sample. However, an attacker may not be able to remove the users they already know from a dataset and, instead, risks wasting guesses retrieving data they already know.

First, in Section 7.2, we discuss the graphs that show sampling with replacement. When guessing with replacement, we simply rank and order our samples and use the ordering to guess the passwords in the dataset. We mark how many users we managed to compromise and subtract this from the optimum number of users we could have compromised had we used the best choice of password for that guess number, as per the function $H_{q^{n}}\left(g\right)$. This loss value is plotted as a function of the number of guesses.

Second, in Section 7.3, guessing without replacement removes the bias from marking a success when the user’s password in question was already part of our sample. Our method for guessing without replacement involves sampling 1000 passwords from each dataset without replacement. These samples are then used to guess the passwords in each dataset. However, when guessing using a sample, *x*, chosen from the dataset we are now guessing, *X*, we remove all the users who were used to form our sample from the dataset; X−x. Thus, we are now using *x* to try to guess X−x. All samples that were chosen from a different dataset can be used to guess dataset *X* as normal.

### 7.2. Guessing Results: With Replacement

**Figure 10:**
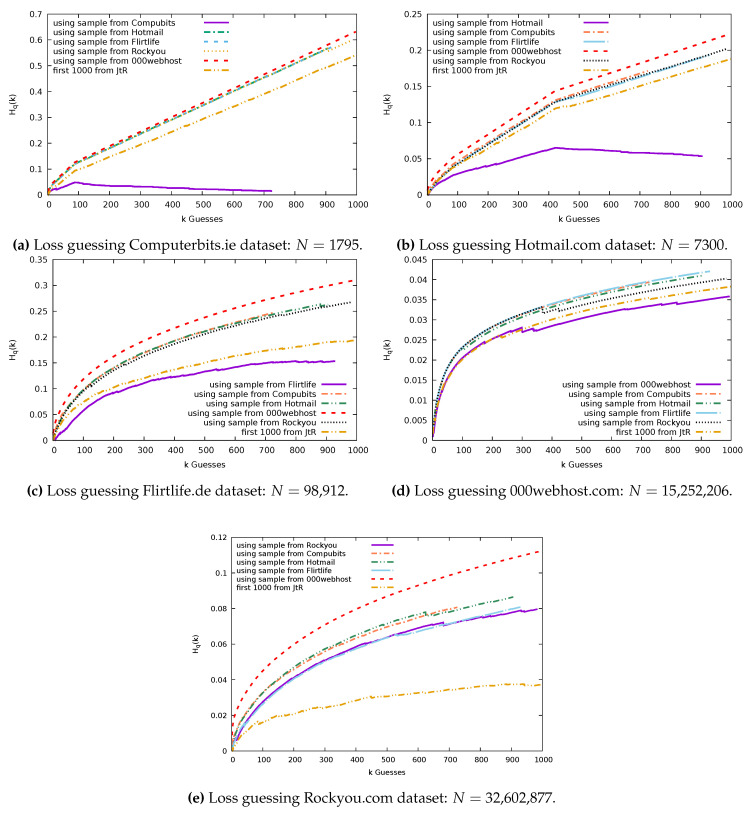
Datasets guessed using n=1000 samples from Computerbits, Hotmail, Flirtlife, 000webhost, Rockyou, and John the Ripper (JtR) with replacement.

Figure 10 shows the comparisons of guessing effectiveness when sampling with replacement for each sample source. Notice that, depending on the sample source, there are a different number of guesses made. For example, in Figure 10a, the sample from Computerbits made just 726 guesses, whereas the Rockyou sample made 984 guesses. This is because, when we sampled 1000 users from the Computerbits dataset, we returned only 726 distinct passwords. We can use the expectation of the number of distinct passwords to estimate the number of distinct passwords we actually see in a sample of size *n*:

$$\sum_{i}\left(1-{\left(1-p_{i}\right)}^{n}\right)$$

Using this, we expect to see 732 distinct passwords in a sample of 1000 users’ passwords from the Computerbits dataset, 915 in a Hotmail sample, 909 for Flirtlife, 995 in a 000webhost sample, and 979 in a Rockyou sample. This is a reflection of the proportion of the probability that lies in the high frequency passwords in each dataset.

In Figure 10a we see that the Computerbits sample is by far the most effective at guessing the Computerbits dataset. It offers improvements over guessing with the basic JtR wordlist or with samples from the other four datasets. A turning point occurs at g=88, when we guess with the sample from Computerbits. This is because all passwords of rank greater than g=88 have frequency 1.

Figure 10b shows that the Hotmail sample guesses the Hotmail dataset most effectively. We can also see that the distance from the optimum begins decreasing from g=420 guesses, where we encounter the passwords that occur with frequency 1. For both the Hotmail dataset and the Computerbits dataset, the JtR basic wordlist performs only slightly more effectively than guessing with the samples of passwords collected from the other datasets.

Figure 10c depicts the Flirtlife sample guessing the Flirtlife dataset the most effectively. The JtR list performs more effectively than all but the Flirtlife sample.

In Figure 10d, we can see that the sample chosen from the 000webhost dataset guesses the 000webhost dataset most effectively. In every other plot, the 000webhost sample is significantly worse than all other samples when used for guessing. These is likely a consequence of the composition rules that the 000webhost website enforced; while the other sites enforced no rules, 000webhost passwords must include both letters and numbers.

The John the Ripper set does a good job of guessing the 000webhost dataset, and the Rockyou sample is the next best option.

Figure 10e shows the Rockyou password set guessed using each of our five samples. The 000webhost sample is least effective at guessing the Rockyou dataset. This is followed by the Computerbits and Hotmail samples. There is very little difference between the returns from the Flirtlife sample and the Rockyou sample. However, when repeated for 100 trials, we find that the mean loss is higher for the Flirtlife sample; therefore, the Rockyou samples do overall perform better. Surprisingly, the John the Ripper wordlist outperforms the Rockyou sample. The Rockyou dataset is a wordlist option for the John the Ripper tool. It is likely that the Rockyou password distribution was used to form the contents and ordering of the passwords in the John the Ripper basic wordlist we are using. We suspect this explains why it can so effectively guess the Rockyou data and outperforms a sample of 1000 Rockyou users.

### 7.3. Guessing Results: Without Replacement

**Figure 11:**
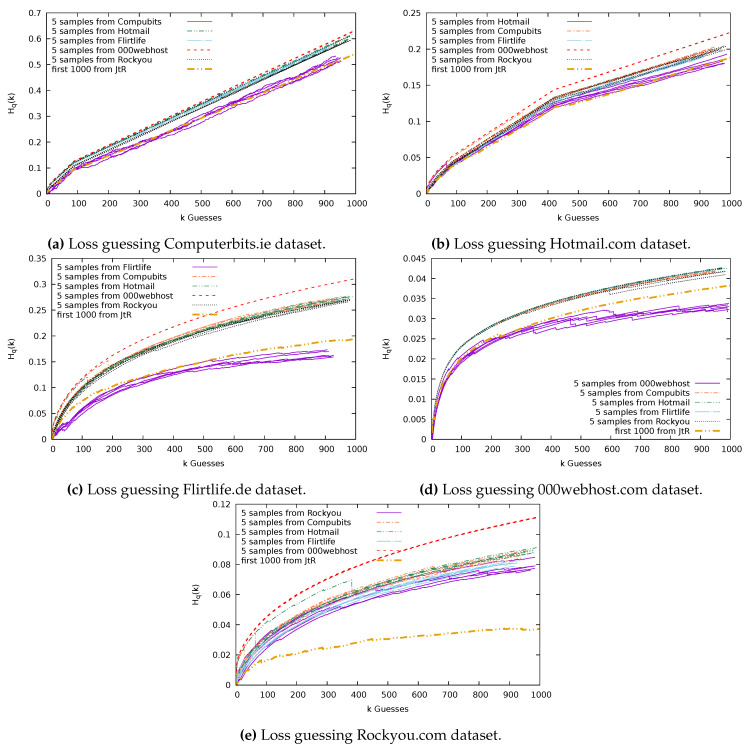
Datasets guessed using samples, of size n=1000, chosen without replacement from Computerbits, Hotmail, Flirtlife, 000webhost, Rockyou, and JtR.

Now, we report the results of our guessing without replacement. Again, we will find that when guessing using the sample chosen from the dataset, we are guessing most effectively. In this case, there is less of a spread between the results from using the samples from different datasets. Therefore, in Figure 11, we show the results of 5 guessing trials for each sample. The results are generally much closer to the results of guessing using the established John the Ripper basic wordlist. We will discuss each graph in Figure 11 individually.

Figure 11a shows that the sample from Computerbits guesses the remaining users’ passwords in Computerbits with less loss than guessing with samples from Rockyou, 000webhost, Flirtlife, or Hotmail. However, though Computerbits offers the best guessing, the loss as a result of guessing the Computerbits dataset is very high (<∼0.6) in comparison to the loss when guessing the other four datasets (<∼0.3).

In Figure 11b, again, we see that guessing with the sample from the Hotmail dataset offers more successful guessing of Hotmail than guessing with the samples from the other datasets. Similar to the situation for the other datasets, the 000webhost sample gives the worst returns when used to guess the Hotmail dataset.

In Figure 11c, the sample from Flirtlife performs best at guessing Flirtlife, showing significant improvements over guessing with the other samples and over guessing with the JtR basic wordlist.

Figure 11d shows the sample from 000webhost guessing the remaining users’ passwords in 000webhost better than the other samples and JtR. In fact, we can see that the loss when guessing 000webhost passwords is very low. For the 000webhost sample, we get a final loss of less than 0.035. That is, we failed to compromise just 3.5% of the users we could have optimally compromised in 1000 guesses. Even for guessing using the other datasets, we had a loss of less that 0.045 when guessing 000webhost.

Similar to when we guessed the Rockyou dataset using samples chosen with replacement, in Figure 10e, we again find that guessing using a Rockyou sample and a Flirtlife sample offer similar results. Running 100 trials, we get a 90% confidence interval for the end loss as a result of guessing Rockyou using samples from Rockyou of [0.0770, 0.083], and a 90% confidence interval for samples drawn from Flirtlife gives a loss interval of [0.0774, 0.083]. These are close, but one thing to note is that the loss is consistently increasing with each guess made. The Rockyou samples can make an average of 979 guesses, whereas Flirtlife samples make an average of only 912 guesses. In Figure 11e, we see that at the end of the guessing the Flirtlife loss stops increasing, whereas the Rockyou loss continues to increase for the additional guesses it makes, making the end result of the loss from guessing higher in comparison for Rockyou.

## 8. Discussion

In Figure 11a, we find that a sample from Computerbits guesses the remaining users’ passwords in Computerbits better than any of the samples from Rockyou, 000webhost, Flirtlife, or Hotmail. This is interesting because if we assume all users choose passwords with a certain distribution, then we would expect Rockyou with 32 million users’ passwords to give the best approximation of that distribution. Instead, Computerbits, with just 1795 users, offers the best guessing success. This tells us that there are certain characteristics within a dataset, such as demographic of users and inclusion of site-specific terminology, that impact choice of passwords. This supports the work of other researchers [14,15] and confirms that access to a small number of users’ passwords from a particular website can be leveraged to compromise remaining users of the same site.

We have shown both theoretically and in practice that a sample of passwords taken from a dataset will help compromise the remaining users in that dataset. This research provides evidence supporting the advice: “Users should be prohibited from creating passwords obtained from previous breaches” [2,26]. Our findings also highlight the importance of protecting every user of a website or online service. Sometimes, a small number of users can be compromised by phishing, guessing attacks, or other means, but, by proof of convergence of this sample towards the distribution of the whole dataset, we know that the information gained by the attackers about the passwords of these users can be used to compromise the remaining users in the dataset.

We observed that samples fromm the 000webhost dataset were less effective for guessing the other datasets we considered. It seems likely that this means that one should consider composition policy when doing cross-website password guessing. This suggests that, while passwords from the same source are most likely to compromise users, sources of passwords with matching composition policies are more likely to produce matches.

When guessing the 000webhost dataset, we found that we were able to guess with very little loss. Figure 11d shows that, using 1000 users to guess the 000webhost dataset results in loss less than 0.045, whereas guessing the Rockyou password set (Figure 11e) results in a loss of up to 0.12. Looking at the frequency with which passwords are chosen can give us some insight into why the loss for 000webhost is low. The 10 most popular passwords in the Rockyou dataset account for 2.05% of all the Rockyou users’ passwords, whereas the 10 most popular 000webhost passwords account for 0.8% of the users’ passwords. We conjecture that the introduction of the composition policy reduces the non-uniformity of the password distribution.

In 2016, Florêncio, Herley, and Van Oorschot pointed out that, once a few accounts are compromised, control over these can be leveraged to bring down a whole system. They introduced the concept of a saturation point, $\alpha_{sat}$, a value between 0 and 1 which represents the fraction of the user population that needs to be compromised before the entire system can be said to be overrun. The authors suggest that, once α = 0.5, a system can be considered to be completely overrun. At this point, there would be very few resources the attacker cannot access, and, due to malicious actions from the compromised accounts, such as spam, the system would become unusable. Demonstrated why in this paper, as well as explicitly stated in our previous paper [1], if we have a sample which is 10% of the users in the Flirtlife dataset, then we can leverage the passwords in this sample to compromise approximately 45% of the remaining users in the Flirtlife set. Thus, jumping our number of compromised accounts much closer to the saturation point.

We are impressed by how well the basic John the Ripper wordlist does as guessing. When guessing without replacement, the basic JtR wordlist, at times, performs well at guessing the Computerbits and Hotmail datasets and is consistently best at guessing the Rockyou dataset. The strength of JtR lies in mangling rules. We believe this work provides evidence for the power of seeding JtR input wordlists with passwords gained from previous small leaks from the same website. Mangling rules can also then be applied to these inputs.

## 9. Conclusions

In this paper, we introduced a model for measuring the effectiveness of password guessing. Leveraging this model, we proved that, by using a sample of passwords, we can capably guess the password in a dataset with a loss which converges to zero as the number of samples gets large. We also provided variations of these proofs which allow for a small amount of guessing loss and, therefore, more accurately describe the empirical data and the motivations of an attacker. Our guessing model is used to visualize the effectiveness of samples at guessing and allows us to compare the performance of different samples at guessing datasets of different sizes. We demonstrated that organizations face the risk of subsequent compromises when a subset of their users’ passwords are revealed. We have shown both theoretically and in practice that a sample of passwords taken from a dataset will help compromise the remaining users in that dataset effectively.

## Figures and Tables

**Table 1:** Spread of loss results for 20 samples of each size *n* which are guessing of the Flirtlife dataset.

Sample Size		Spread of Loss			
% of N	n	Mean	max	min	Range
1%	989	15151.0	16608	14487	2121
10%	9891	12651.3	12980	12390	590
25%	24728	9194.9	9394	8946	448
50%	49456	4109.6	4219	3987	232
75%	74184	2100.1	2181	2018	163
100%	98912	1139.1	1210	1073	137
200%	197824	122.7	136	106	30

**Table 2:** First 10 passwords in the John the Ripper wordlist.

Rank	Password
1	123456
2	12345
3	password
4	password1
5	123456789
6	12345678
7	1234567890
8	abc123
9	computer
10	tigger

## Abbreviations

The following abbreviations are used in this manuscript:

JtR	John the Ripper password cracking software
CLT	Central Limit Theorem
